# The effect of enhanced recovery after surgery on the risk factors of venous thromboembolism for patients with gynecologic malignancies

**DOI:** 10.3389/fonc.2025.1627605

**Published:** 2025-11-10

**Authors:** Jie Xu, Shanshan Liu, Dan Liu, Yina Wang, Meilan Tang

**Affiliations:** Obstetrics and Gynecology Department of Yancheng Third People’s Hospital, The Yancheng School of Clinical Medicine of Nanjing Medical University, Yancheng, China

**Keywords:** enhanced recovery surgery, gynecologic malignancies, venous thromboembolism, risk factors, adverse events

## Abstract

**Objective:**

This study aims to evaluate the efficacy of enhanced recovery after surgery (ERAS) in mitigating the risk of venous thrombosis in patients undergoing surgery for gynecological malignancies.

**Methods:**

This prospective randomized controlled trial enrolled patients from January 2019 to December 2022, who were randomly assigned to either the experimental group (ERAS management) or a control group (conventional treatment). The primary endpoints were perioperative venous thrombosis risk indicators, while secondary outcomes involved the incidence of venous thromboembolism (VTE) events and other clinically relevant adverse events.

**Results:**

A total of 177 patients were included, with 91 in the experimental group and 86 in the control group. Preoperative characteristics were comparable between the groups (P>0.05). At one-week post-surgery, the experimental group exhibited higher hemoglobin levels and lower white blood cell counts, D-dimer values, and proportions of patients classified as high risk for thrombosis compared to the control group (P<0.05). Additionally, the incidence of VTE events was significantly lower in the experimental group one month post-surgery (P<0.05).

**Conclusion:**

The implementation of ERAS significantly reduces perioperative venous thrombosis risk in patients with gynecological malignancies, demonstrating both safe and effective.

## 1 Introduction

Venous thromboembolism (VTE), which includes deep vein thrombosis (DVT) and pulmonary embolism (PE), is a serious complication in patients with malignancies (1). Research shows that patients with gynecological cancers are at a significantly higher risk of thrombosis compared to those with other tumors (2, 3), likely because these tumors are confined to the pelvis, facilitating early development of lower extremity DVT. Without preventive measures, postoperative DVT can occur in up to 26% of patients, and PE in up to 9% of those with gynecological malignancies (4), making VTE one of the most lethal complications of gynecologic cancer surgery (5). However, due to the specific pelvic anatomy involved in gynecological surgery, there is also a considerable risk of major postoperative bleeding (6). Weighing the benefits and risks of thromboprophylaxis, perioperative guidelines for thromboembolism prevention in gynecological oncology patients remain under active development, and clinical evidence in this area is still insufficient.

Enhanced Recovery After Surgery (ERAS) is an evidence-based, multidisciplinary approach designed to optimize perioperative care, reduce complications and surgical stress, and accelerate recovery (7). First introduced by Henrik Kehlet in 1997, it is now widely applied in various surgical specialties, including gynecology (8). Key components of ERAS include: preoperative management (e.g., patient education, nutritional support, smoking and alcohol cessation); intraoperative measures (e.g., anesthesia protocols, antibiotic prophylaxis, temperature management); and postoperative strategies (e.g., pain control, early oral intake, mobilization, and fluid management). Discharge criteria and follow-up are also standardized.

Accumulating evidence indicates that ERAS can reduce the incidence of VTE compared to conventional perioperative care (9, 10). For example, Li et al. (11) reported that an ERAS protocol significantly reduced VTE risk in patients receiving first-line therapy for advanced disease—only 1 of 46 patients experienced VTE within 30 days post-surgery, and the 6-month cumulative incidence was 6.1%. However, more evidence is needed to clarify the role and refine the application of ERAS in preventing venous thrombosis among gynecological tumor patients. Therefore, this study focuses on patients undergoing surgery for gynecological malignancies, with the aim of evaluating the impact of an ERAS program on perioperative venous thrombosis risk and providing a reference for clinical practice.

## 2 Materials and methods

### 2.1 Study population and group allocation

Patients with gynecological malignant tumors who underwent surgical procedures in our hospital within the period from January 1, 2019, to December 30, 2022, were consecutively enrolled and randomly assigned to either the experimental group or the control group. The experimental group was subjected to ERAS management, whereas the control group received conventional management. This study was duly approved by the Medical Ethics Committee of our hospital.

The inclusion criteria were as follows: (1) Age ranging from 18 to 75 years; (2) Diagnosis of gynecological malignant tumor established through clinical or pathological examination; (3) Clinical stage conforming to the indications for tumor surgery; (4) Voluntary signing of the informed consent for surgery by the patients.

The exclusion criteria encompassed: (1) Complicated with severe underlying medical conditions; (2) Undergoing preoperative chemoradiotherapy; (3) Previous history of thromboembolic events or hematological disorders; (4) Long-term use of contraceptives, non-steroidal drugs, or anticoagulants.

The withdrawal criteria consisted of: (1) Voluntary withdrawal by the patient; (2) Occurrence of serious adverse events; (3) Loss to follow-up.

The study conducted single-blind design, wherein patients were unaware of their group allocation while healthcare providers were not blinded. To address the potential risk of bias, radiologists, outcome assessors, and data analysts were blinded to the group assignments in this study. The radiologists performing and interpreting the imaging examinations were unaware of the patients’ group allocation. Rigorous adherence to the random allocation process was maintained to ensure the baseline comparability of the two groups, enhancing the internal validity of the study and the reliability of the observed results.

### 2.2 Methodology

The conventional procedure for control group: routine preoperative education was provided, intraoperative fluid infusion was carried out conventionally, the operating environment was maintained at normal temperature, an analgesic pump was installed at the patient’s request at the end of the operation, liquid intake was initiated after anal exhaust, preventive anticoagulation medication was administered 24 hours postoperatively, and ambulation was encouraged 1 to 2 days after the operation.

The ERAS procedure for experimental group:

1. Preoperative Prehabilitation Protocol: Anemia, obesity, and anxiety symptoms were optimized through dietary adjustments, pharmacological interventions, and psychological counseling 2–4 weeks prior to admission. Low molecular weight heparin was administered for thrombosis prevention 1 hour before surgery (8).
2. Intraoperative Management: Fluid infusion volume was individualized and controlled, and a heating blanket was utilized to regulate body temperature.
3. Postoperative Care: Early enteral nutrition was initiated 6 hours after the operation. Low molecular weight heparin was administered 12 hours postoperatively in combination with mechanical methods (pneumatic compression device or elastic stocking). Ambulation was commenced within 24 hours after the operation.
4. Follow-up: Laboratory examinations and thrombosis risk assessments were conducted in the outpatient clinic at 1 week and 6 month after the operation. Patients who were lost to follow-up were excluded from the analysis.

Both groups of patients were operated on by highly qualified chief physician teams. The specific surgical procedures included abdominal or laparoscopic radical hysterectomy, accompanied by salpingectomy and/or ovariectomy, and pelvic lymphadenectomy. Peripheral venous blood samples were collected for detection in a fasting state.

### 2.3 Evaluation metrics

1. The primary outcome was centered around the perioperative venous thrombosis risk indicators for both groups. This incorporated laboratory assays and the quantitative thrombosis risk score designed specifically for cancer patients. The Khorana Score (KS), recommended by the guidelines of the American Society of Clinical Oncology (ASCO) and the European Society for Medical Oncology (ESMO) and validated through multiple research studies, was employed for quantitative scoring. The scoring criteria encompassed tumor type (with gynecologic neoplasms assigned 1 point), along with the following clinical parameters: hemoglobin level < 100 g/L (1 point), platelet count ≥ 350 × 10^9^/L (1 point), white blood cell count > 11.0 × 10^9^/L (1 point), and body mass index (BMI) ≥ 35 kg/m² (1 point). The calculated scores were denoted as the KS value, and the risk stratification was as follows: high risk (KS value ≥ 3), intermediate risk (KS value = 1 or 2), or low risk (KS value = 0). Additionally, prior investigations have demonstrated that D-dimer can serve as an adjunct in the diagnosis of venous thromboembolism and in predicting the recurrence risk of venous thromboembolism (12, 13).
2. The secondary outcomes focused on adverse events, such as the incidence of venous thromboembolism episodes. The diagnostic criteria were established based on a combination of clinical assessment and definitive imaging confirmation, in accordance with standard clinical guidelines (14). DVT was suspected in patients presenting with clinical signs such as unilateral limb swelling, pain, warmth, and erythema. The diagnosis was then definitively confirmed by color Doppler ultrasound compression examination, which is the primary and preferred initial imaging modality for suspected DVT. Pulmonary embolism (PE) was suspected in cases of sudden-onset chest pain, tachypnea, tachycardia, cough, or dyspnea. The diagnosis was verified by spiral CT pulmonary angiography (CTPA), which is the imaging gold standard for confirming PE.

### 2.4 Statistical analysis

SPSS 26.0 software was utilized for the statistical analysis of the full analysis set. The measurement data were expressed as the mean ± standard deviation ( $\bar{\text{x}}$ ± s), and the independent samples t-test was applied for inter-group comparisons. The count data were presented as rates (%), and the chi-square (χ²) test or Fisher’s exact test was used for group comparisons. For ranked data, the Wilcoxon rank sum test was employed for inter-group comparisons. A p-value less than 0.05 was considered statistically significant.

## 3 Results

### 3.1 General characteristics of clinical data

**Figure 1:**
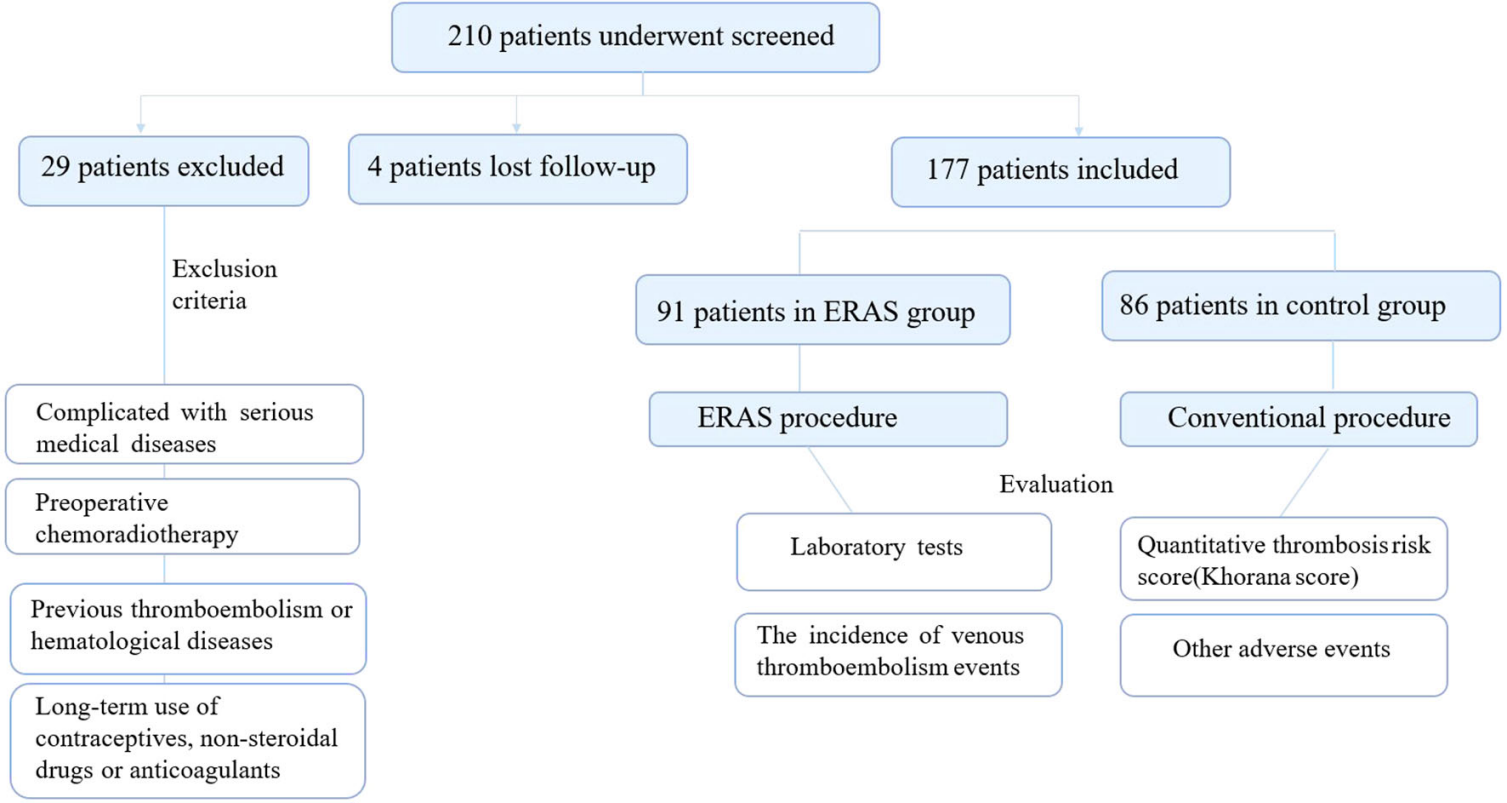
Study flow diagram.

During the period from January 2019 to December 2022, a total of 342 patients were initially screened. Of these, 27 patients did not fulfill the inclusion criteria. Eventually, 315 patients were incorporated into the final analysis, with 161 patients assigned to the experimental group (undergoing ERAS management) and 154 patients to the control group (receiving conventional treatment) (**Figure 1**).

There were no statistically significant discrepancies between the two groups with respect to age, body mass index (BMI), tumor type, tumor stage, and surgical approach (P>0.05), thereby ensuring the comparability of the two cohorts. Refer to **Table 1** for detailed information.

**Table 1:** Comparison of general clinical data characteristics between the ERAS group and control group.

Characteristic	Items	ERAS group (N = 161)	Control group (N = 154)	Statistical values	P-value
Age(years)		56.0 ± 8.9	54.8 ± 9.5	1.405	0.237
BMI (Kg/m2)		23.4 ± 2.5	23.0 ± 2.5	1.887	0.171
Source of malignancy (n)	Uterus Lining	52	49	0.067	0.967
	eggs The nest	19	17	-	-
	palace neck	90	88	-	-
Tumor stage (n)	I period	71	78	Fisher	0.501
	II period	50	38	-	-
	III period	35	35	-	-
	stage IV	5	3	-	-
Surgical approach (n)	Open surgery	145	131	1.812	0.178
	Laparoscopic surgery	16	23		
surgical type (n)	Radical hysterectomy	161	154	0.105	0.746
	With bilateral or unilateral salpingo-oophorectomy	149	141		
	Without ovaries	12	13		
	Lymph node dissection	135	140	3.537	0.06
	Removal of omentum	19	17	0.045	0.832

BMI is the body mass index, the square of weight/height (international units kg/square meter).

### 3.2 Perioperative venous thrombosis risk laboratory indexes comparison

No substantial alterations in BMI were detected pre- and post-surgery within either of the two groups. Regarding the preoperative values of hemoglobin (HB), platelet count (PLT), white blood cell count (WBC), and D-dimer (D-D), no significant differences were noted (P>0.05). At one week following the surgical procedure, both groups exhibited a decrease in HB levels and an increase in PLT, WBC, and D-D levels. However, the experimental group demonstrated significantly elevated HB levels and substantially lower WBC and D-D values in comparison to the control group (P<0.001). Conversely, the difference in PLT values between the two groups did not reach statistical significance (P>0.05). Refer to **Table 2** for comprehensive data.

**Table 2:** Comparison of perioperative laboratory measures of tumor thrombosis risk between the eras group and control group.

Characteristics	Stage	ERAS group (N = 161)	Control group (N = 154)	T-score	P-value
HB(g/l)	Pre-operative	119.6± 13.5	120.1± 14.5	0.085	0.771
	Post-operative	108.0 ± 14.3	101.4± 18.0	13.116	< 0.001
PLT(×10^9^/l)	Pre-operative	255.3 ± 31.5	261.3 ± 36.1	2.487	0.116
	Post-operative	290.3 ± 38.5	296.9± 41.0	2.353	0.126
WBC(×10^9^/l)	Pre-operative	9.6 ± 2.4	9.8 ± 1.7	1.183	0.277
	Post-operative	10.6 ± 2.3	12.0 ± 2.1	30.079	< 0.001
D-D(mg/l)	Pre-operative	0.59 ± 0.20	0.60 ± 0.19	0.014	0.905
	Post-operative	3.20 ± 0.50	4.60 ± 0.80	364.258	< 0.001

### 3.3 Khorana score comparison

Khorana Score (KS) is used for evaluating the tumor thrombosis risk, which means high risk (KS value ≥3), medium risk (KS value =1 or 2) or low risk (KS value =0). The proportion of patients with a high-risk (KS ≥ 3) in the experimental group was markedly lower than that in the control group at postoperative assessment (P<0.001), while no statistically significant difference was observed preoperatively (P>0.05). Refer to **Table 3** for detailed breakdown.

**Table 3:** Comparison of Khorana score for perioperative tumor thrombosis risk between the control group and ERAS group.

Characteristics	Khorana score	ERAS group (N = 161)	Control group (N = 154)	Z-score	P-value
Pre-operative	KS <3 points	155 (96.3)	142 (92.2)	2.415	0.12
	KS≥3 points	6 (3.7)	12 (7.8)		
Post-operative	KS <3 points	135 (83.9)	99 (64.3)	15.773	<0.001
	KS≥3 points	26 (16.1)	55 (35.7)		

### 3.4 Postoperative venous thrombosis and other adverse events

#### 3.4.1 Venous thrombosis incidence

Over the six-month follow-up interval, a significant disparity in the occurrence of venous thromboembolism was noted between the two groups. In the control group, seven cases of VTE were observed, corresponding to an incidence rate of 4.5% (7/154). In the experimental group, one case was recorded (0.6%, 1/161), and this difference attained statistical significance (P = 0.033).

#### 3.4.2 Analysis of other adverse events

In addition to thrombosis, the incidence of other adverse events was also monitored. The number of febrile episodes was 16 in the experimental group and 19 in the control group. For infectious complications, the figures were 4 in the experimental group and 6 in the control group. Regarding bleeding events, 5 cases were recorded in the experimental group and 3 in the control group. Statistical analysis, employing appropriate tests, determined that these differences did not reach statistical significance (P>0.05) (**Table 4**).

**Table 4:** Comparison of VTE incidence and other adverse events for between the control group and ERAS group.

Items		ERAS group (N = 161)	Control group (N = 154)	P-value
VTE incidence		1	7	0.033
other adverse events	febrile episodes	16	19	0.498
	infection	4	6	0.534
	bleeding	5	3	0.724

#### 3.4.3 Methods revision

Patients with gynecological malignant tumors who underwent surgical procedures in our hospital within the period from January 1, 2019, to December 30, 2022, were consecutively enrolled and randomly assigned to either the experimental group or the control group. The experimental group was subjected to ERAS management, whereas the control group received conventional management. This study was duly approved by the Medical Ethics Committee of our hospital.

## 4 Discussion

Our study adopted a quantitative classification methodology to assess the risk of venous thromboembolism in patients with gynecological cancer during the perioperative period. It delved into the impact of ERAS on venous thrombosis from multiple aspects, including laboratory indicators, risk assessment levels, and the incidence of thrombotic events. The results indicated no conspicuous differences in preoperative general information and risk indicators between the two groups. However, significant alterations were observed postoperatively. During the perioperative phase, due to blood and fluid losses plus surgical stress and inflammatory mechanisms, an inevitable decline in hemoglobin values occurred, accompanied by elevated white blood cell count, platelet count, and higher D-dimer levels, all signaling a hypercoagulable state and augmented thrombosis risk (13). In the ERAS group, the trends of changes in hemoglobin, white blood cell count, D-dimer values, KS risk grading, and the proportion of high-risk patients were more favorable. This suggests these patients had a lower probability of experiencing anemia, inflammatory stress, and hypercoagulability, potentially leading to reduced VTE risk. Notably, the VTE incidence was significantly lower in the ERAS group, highlighting the efficacy of comprehensive thromboprophylaxis within ERAS.

Surgery remains a primary treatment for gynecological malignancies, and both the cancers and specific pelvic surgeries are established high-risk determinants for VTE (1). VTE increases in-hospital mortality, impairs quality of life, and raises medical resource consumption (11). Recurrent thrombosis may necessitate extended or lifelong anticoagulation, concomitantly increasing bleeding risk. Therefore, implementing safe and effective perioperative thrombosis prevention is crucial. ERAS has shown significant benefits in recovery for various diseases, and efforts to integrate it into gynecological tumor management are ongoing (15, 16).

Our findings extend the evidence that ERAS reduces VTE occurrence in gynecologic tumors. Several studies corroborate the effectiveness of ERAS protocols in reducing VTE. For example, Wijk et al. reported that ERAS in gynecologic surgery significantly reduced postoperative complications, with a notable decline in VTE incidence, closely resembling our results (17). Similarly, Nelson et al. emphasized that preoperative optimization and early mobilization, key ERAS components, are pivotal in minimizing thromboembolic risk (18). This concordance demonstrates that ERAS can substantially enhance patient outcomes against VTE. Simultaneously, trials like ERAS GYN are exploring optimal anticoagulation duration and its correlation with thromboprophylaxis in the ERAS context (14).

The ERAS guidelines applied here advocate for initiating thromboembolism prophylaxis before admission, including pre rehabilitation, plus meticulous intraoperative fluid and temperature management, and optimizing conditions like anemia and obesity. These measures aim to reduce transfusion needs and enhance recovery. Anemia, a known risk factor, predisposes patients to transfusions and complications; ERAS guidelines recommend not proceeding with elective surgery without corrective treatment (19). Obesity is another significant risk factor, associated with worse prognosis and more complications; preoperative weight loss has been shown to improve the postoperative course (20). Intraoperative fluid management seeks equilibrium, as hypovolemia risks tissue hypoxia and hypervolemia may cause edema (21). Maintaining normothermia is also emphasized to avoid surgical site infections. Early postoperative mobilization, such as ambulation within 24 hours, helps prevent complications including pulmonary infection, VTE, and intestinal obstruction. Preemptive low molecular weight heparin use is recommended, extended to 28 days for high risk patients. Collectively, these perioperative measures modulate venous flow, attenuate surgical stress and inflammatory injury, and facilitate muscle activity, thereby reducing thromboembolism incidence.

ERAS offers a comprehensive perioperative approach that improves venous flow, mitigates surgical stress and vascular damage, and through promoting limb movement, diminishes thromboembolism. It serves as a valuable principle for VTE prevention in gynecological malignancies, reducing complications without adding significant risks or economic burdens.

However, this study has limitations. The most prominent is the absence of double blinding, potentially introducing performance bias affecting care quality and patient recovery perceptions. Also, although randomization was effective, more elaborate randomization or stratification could have enhanced group comparability. These factors may affect the reproducibility and generalizability of the results.

Future research should incorporate double blind designs and consider stratified randomization to minimize biases and improve comparability. Addressing these aspects will allow future studies to build on this foundation and further refine ERAS application in gynecological oncology, improving patient care and recovery outcomes.

## 5 Conclusion

In general, this research demonstrated that the implementation of ERAS significantly reduces perioperative venous thrombosis risk in patients with gynecological malignancies effectively. Our research endeavors to refine the existing perioperative management strategies, with a focused effort on bridging the knowledge gaps, especially when dealing with high-risk patient populations. By undertaking such initiatives, we offer promising avenues for future research directions and hold the potential to revolutionize the standard of care in the realm of gynecological cancer management.

## Data Availability

The original contributions presented in the study are included in the article/Supplementary Material. Further inquiries can be directed to the corresponding author.

## References

[1] Donnellan E Khorana AA . Cancer and venous thromboembolic disease: a review. Oncologist. (2017) 22:207. doi: 10.1634/theoncologist.2016-0214, PMID: 28174293 PMC5330704

[2] Mokri B Mariani A Heit JA Weaver AL McGree ME Martin JR . Incidence and predictors of venous thromboembolism after debulking surgery for epithelial ovarian cancer. Int J Gynecol Cancer. (2013) 23:1684–91. doi: 10.1097/IGC.0b013e3182a80aa7, PMID: 24172104 PMC4307403

[3] Abu Saadeh F Norris L O'Toole S Gleeson N . Venous thromboembolism in ovarian cancer: incidence, risk factors and impact on survival. Eur J Obstet Gynecol Reprod Biol. (2013) 170:214–8. doi: 10.1016/j.ejogrb.2013.06.004, PMID: 23830352

[4] Clarke-Pearson DL Abaid LN Prevention of venous thromboembolic events after gynecologic surgery. Obstet Gynecol. (2012) 119:155–67. doi: 10.1097/AOG.0b013e31823d389e, PMID: 22183223

[5] Guntupalli SR Brennecke A Behbakht K Tayebnejad A Breed CA Babayan LM . Safety and efficacy of apixaban vs enoxaparin for preventing postoperative venous thromboembolism in women undergoing surgery for gynecologic Malignant neoplasm: a randomized clinical trial. JAMA Netw Open. (2020) 3:e207410. doi: 10.1001/jamanetworkopen.2020.7410, PMID: 32589230 PMC7320298

[6] Watrowski R Jager C Forster J . Improvement of perioperative outcomes in major gynecological and gynecologic-oncological surgery with hemostatic gelatin-thrombin matrix. In Vivo. (2017) 31:251–8. doi: 10.21873/invivo.11053, PMID: 28358708 PMC5411753

[7] Wijk L Udumyan R Pache B Altman AD Williams LL Elias KM . International validation of Enhanced Recovery After Surgery Society guidelines on enhanced recovery for gynecologic surgery. Am J Obstet Gynecol. (2019) 221:237.e1–237.e11. doi: 10.1016/j.ajog.2019.04.028, PMID: 31051119

[8] Garcia Erce JA Laso Morales MJ . Patient blood management in the enhanced recovery program after abdominal surgery. Cir Esp. (2017) 95:552–4. doi: 10.1016/j.ciresp.2017.02.001, PMID: 28318496

[9] Schneider S Armbrust R Spies C du Bois A Sehouli J . Prehabilitation programs and ERAS protocols in gynecological oncology: a comprehensive review. Arch Gynecol Obstet. (2020) 301:315–26. doi: 10.1007/s00404-019-05321-7, PMID: 31616986

[10] Khorana AA Kuderer NM Culakova E Lyman GH Francis CW . Development and validation of a predictive model for chemotherapy-associated thrombosis. Blood. (2008) 111:4902–7. doi: 10.1182/blood-2007-10-116327, PMID: 18216292 PMC2384124

[11] Li S Bercow AS Falzone M Kalyanaraman R Worley MJ Feltmate CM . Risk of venous thromboembolism for ovarian cancer patients during first-line therapy after implementation of an Enhanced Recovery After Surgery (ERAS) protocol. Gynecol Oncol. (2021) 162:353–9. doi: 10.1016/j.ygyno.2021.05.032, PMID: 34092412

[12] Lyman GH Bohlke K Khorana AA Kuderer NM Lee AY Arcelus JI . Venous thromboembolism prophylaxis and treatment in patients with cancer: American Society of Clinical Oncology clinical practice guideline update 2014. J Clin Oncol. (2015) 33:654–6. doi: 10.1200/JCO.2014.59.7351, PMID: 25605844 PMC4881372

[13] Patell R Rybicki L McCrae KR Khorana AA . Predicting risk of venous thromboembolism in hospitalized cancer patients: utility of a risk assessment tool. Am J Hematol. (2017) 92:501–7. doi: 10.1002/ajh.24700, PMID: 28240823 PMC5729904

[14] Xu J Han HC Chong QG Wang H . The significance of plasma D-dimer and platelet levels in Malignant tumors with early manifestation of VTE. Chin J Ophthalmol. (2019) 21:596–8. doi: 10.3760/cma.j.issn.1008-1372.2019.04.030

[15] Su M . Effect of enhanced recovery after surgery on prevention of deep vein thrombosis in high-risk population after gynecological pelvic surgery. China Pract Med. (2018) 13:182–3.

[16] Scottish Intercollegiate Guidelines Network . Prevention and management of venous thromboembolism: a national clinical guideline (2010). Edinburgh: SIGN. Available online at: https://www.sign.ac.uk/assets/sign122.pdf (Accessed August 9, 2025).

[17] de Groot JJ Maessen JM Slangen BF Winkens B Dirksen CD van der Weijden T . A stepped strategy that aims at the nationwide implementation of the Enhanced Recovery After Surgery programme in major gynaecological surgery: study protocol of a cluster randomised controlled trial. Implement Sci. (2015) 10:106. doi: 10.1186/s13012-015-0298-x, PMID: 26223232 PMC4518652

[18] Kjølhede P Bergdahl O Borendal Wodlin N Nilsson L . Effect of intrathecal morphine and epidural analgesia on postoperative recovery after abdominal surgery for gynecologic Malignancy: an open-label randomised trial. BMJ Open. (2019) 9:e024484. doi: 10.1136/bmjopen-2018-024484, PMID: 30837253 PMC6430030

[19] Stefura T Droś J Kacprzyk A Wierdak M Proczko-Stepaniak M Szymański M . Influence of preoperative weight loss on outcomes of bariatric surgery for patients under the enhanced recovery after surgery protocol. Obes Surg. (2019) 29:1134–41. doi: 10.1007/s11695-018-03660-z, PMID: 30632072

[20] Moningi S Patki A Padhy N Ramachandran G . Enhanced recovery after surgery: an anesthesiologist’s perspective. J Anaesthesiol Clin Pharmacol. (2019) 35:S5–13. doi: 10.4103/joacp.JOACP_238_16, PMID: 31142953 PMC6515715

[21] Graul A Latif N Zhang X Dean LT Morgan M Giuntoli R . Incidence of venous thromboembolism by type of gynecologic Malignancy and surgical modality in the National Surgical Quality Improvement Program. Int J Gynecol Cancer. (2017) 27:581–7. doi: 10.1097/IGC.0000000000000912, PMID: 28187092 PMC5539959

